# A simplified definition of sarcopenia: muscle mass/body weight

**DOI:** 10.1016/j.jnha.2024.100302

**Published:** 2024-06-21

**Authors:** William J. Evans, Luigi Ferrucci

**Affiliations:** a University of California at Berkeley, Berkeley, CA, USA; b National Institute on Aging, Bethesda, MD, USA

The decline of muscle mass and muscle strength is one of the phenotypic characteristics of aging. Although this process can be attenuated by moderate-to-intense physical activity, even individuals that faithfully exercise every day see their muscles shrink and their strength decline. When this process is overt and crosses a critical threshold, it leads to a condition called sarcopenia. Over the last three decades, there has been a titanic effort to define standard criteria for the screening and clinical assessment of sarcopenia. Such efforts were driven by the notion that sarcopenia is a major cause of mobility disability in the elderly, and that screening for and “treating” sarcopenia shall result into mobility loss prevention. Yet, despite dozens of dedicated meetings, workshops, scientific articles, statements from professional societies and even the establishment of an ICD-10 code, no definition of sarcopenia that is widely acknowledged by the scientific community is currently available [1].

The definitions so far attempted are based on three basic variables in various combinations: muscle mass (typically measured by DEXA or computerized tomography as lean mass), muscle strength (either grip or knee extension strength) and lower extremity performance (typically walking speed). All three dimensions present complex assessment and interpretation problems. However, although the initial definition of sarcopenia was straightforward as the age-related loss of skeletal muscle and strength [2,3], there was no method that allowed an accurate measurement of muscle mass. Lean body mass (LBM) was assumed to be an appropriate surrogate assessment of muscle mass and has been so used in several large cohort studies. To date, cross-sectional and longitudinal aging cohort studies have reported little or no relationship between low LBM and increased risk of health-related outcomes, including functional capacity, disability, and mortality [4]. A meta-analysis [5] of longitudinal observational studies in older people (≥65 y) conducted between 1976 and 2012 examined reported data of body composition (BIA, DXA, CT) and physical functional capacity. Although LBM was measured, the authors incorrectly used the term “muscle mass” and concluded that “low muscle mass was not significantly associated with functional decline.” This lack of associated of LBM with functional capacity or health related outcomes has led to a type 2 error in the existing literature and as a result there is no consensus on the role of muscle in defining sarcopenia. This lack of consensus has also resulted in several (>15) ‘consensus’ definitions with no consensus at all [1]. The use of surrogate measures of muscle mass older people fail to distinguish between contractile proteins and other proteins that accumulate in skeletal muscle with aging, such as collagen and amyloid that thicken the matrix between myofibers [6]. Because this phenomenon is widely heterogeneous between individuals, the use of LBM is noisy, not very useful and the lack of relationship between LBM, strength and other health related outcomes more recent ‘consensus’ definitions of sarcopenia use no body composition assessment and rely on function and strength.

New technology that is based on Deuterated Creatine dilution [7], by targeting contractile proteins and their energetic system, is showing promising results to assess this problem. Strength has been proposed to be an appropriate diagnostic measurement of sarcopenia. However, assessing strength is not easy and requires equipment and trained personnel. In addition, the strength required to maintain a good mobility is strongly dependent on the presence of other co-impairments. For example, we recently demonstrated that the energetic cost of walking is significantly higher in people with balance problems, and previous studies found that the strength require to maintaining mobility is higher in people who have significant balance problems [8].

The D_3_Creatine dilution method now allows a non-invasive measurement of total body skeletal muscle mass [9]. This method has been validated in adults of all ages and has been employed in cross-sectional and longitudinal aging studies including the Osteoporotic Fractures in Men (MrOS) Study, Framingham Heart Study, Women’s Healthcare Initiative, Study of Muscle, Mobility and Aging (SOMMA), the Tobago Longitudinal Aging study, and more. Because creatine is actively transported into the sarcomere against a large concentration gradient, almost 98% of the body creatine pool is found in muscle. In addition, creatine and phosphocreatine is co-located with contractile components, potentially providing a measure of the’ functional’ components of skeletal muscle. Studies in the (MrOS) population in which 1425 older, community dwelling men were measured demonstrated that muscle mass assessed with D_3_Cr muscle was strongly and independently associated with strength, functional capacity [10], risk of disability (including IADL), hip fracture [11], and mortality [12]. In addition, a threshold of approximately 25% muscle mass was described for a high risk of a mobility disability defined by chair stand time [13]. For the first time, we now have data demonstrating that longitudinal loss of muscle mass measured by D_3_Cr dilution is significantly associated with decreased strength and walking speed [14]. New data from the MrOs cohort demonstrate a linear relationship between decreasing muscle mass and decreasing strength in very old men over an average of 6 years [15].

We propose a simplified definition of sarcopenia as the term implies (lack of flesh) as low muscle mass. The definitions so far attempted are based on three basic variables in various combinations: muscle mass (typically measured by DEXA of computerized tomography), muscle strength (either grip or knee extension strength) and lower extremity performance (typically walking speed). All three dimensions present complex assessment and interpretation problems. While Low muscle mass directly or indirectly results in several geriatric syndromes, functional parameters such as habitual walking speed and even strength may also result from causes other than muscle mass, per se. For example, Orwoll et al. [16] showed that intramyocellular fat (myosteatosis) is independently (from muscle mass) associated with poor physical performance and adverse outcomes in a cohort of older men. In older men and women, slow habitual walking speed is strongly associated with VO_2peak_ rather than body composition [17]. While many of the physical performance variables that have been included in definitions of sarcopenia may be associated with low muscle mass, they are often more strongly related to variables such as balance, vision, cognitive or neural function.

A simplified definition will have greater clinical utility. At the present time, most physicians have no idea how to diagnose or treat sarcopenia [18], perhaps because of the lack of consensus of what it is or that it is not recognized indication by the Food and Drug Administration for development of sarcopenia drugs. Most health care professionals (HCP) do not routinely measure strength or functional capacity in their older patients. However, if a patient is diagnosed with a mobility disability using a simple questionnaire, a measurement of muscle mass could help determine if the disability is due to low muscle mass or another cause, allowing the health care professional (HCP) to develop a therapeutic plan to eventually treat low muscle mass. A definition of sarcopenia as low % muscle mass will also allow HCPs to determine who may be at risk for several age-associated syndromes that have been associated with low muscle mass and develop a therapy that targets maintenance or improvement in skeletal muscle amount.

The relationship between D_3_Cr muscle mass and functional outcomes is currently being measured in several cohort studies. The data emerging from MrOs, and the Study of Muscle Metabolism and Aging (SOMMA) suggest a critical % muscle mass that is highly associated with mobility disability and is different for men and women. We still do not have any highly effective intervention to counteract sarcopenia, but if we had one, we could define the threshold under which such intervention would result in improvement of physical function. Preservation of muscle mass to combat sarcopenia may prove to be the most effective strategy to preserve independence and face advancing age with dignity. A simplified definition available to all HCPs will go a long way to meet this goal.

## Conflict of interest

WJE is a paid consultant for BioAge Labs and VERU Inc. He works with MyoCorps, Inc. His research in supported by the National Institutes of Health, Muscular Dystrophy Association, Duchenne UK, and Parent Project Muscular Dystrophy.

LF has no competing interests.
